# Postoperative pain in transabdominal preperitoneal laparoscopic hernia repair with staple fixation versus self-fixation mesh

**DOI:** 10.1016/j.heliyon.2024.e30033

**Published:** 2024-04-23

**Authors:** Carlos Eduardo Rey Chaves, Camilo Ramírez-Giraldo, Andrés Isaza-Restrepo, Danny Conde Monroy, Juliana González-Tamayo, Daniela Ayala, Maria Carolina Moreno Matson, Jorge Navarro-Alean

**Affiliations:** a Hospital Universitario Mayor - Méderi. Bogotá, Colombia; b Universidad del Rosario. Bogotá, Colombia; c Pontificia Universidad Javeriana. Bogotá, Colombia

**Keywords:** Laparoscopic, Transabdominal preperitoneal repair, Inguinal hernia, Mesh fixation, Chronic pain

## Abstract

**Background:**

The mesh fixation method is one of the multiple factors associated with chronic postoperative pain in inguinal hernia surgery. The aim of this study is to evaluate postoperative pain associated with the two available fixation strategies (staple fixation versus self-fixating mesh) used in our field.

**Methods:**

We designed an observational study with retrospective cohorts to analyze postoperative pain in patients who underwent a laparoscopic transabdominal preperitoneal inguinal hernia repair with a self-fixating mesh or staple fixation, which are the two available techniques in our field. A total of 296 patients who met the inclusion criteria were included between January 2014 and October 2021.

**Results:**

The evaluated patients’ median age was 66.0 (interquartile range (IQR): 20.75) years and were predominantly male (70.13 %). The proportion of participants with chronic pain was 3.20 % in the staple fixation group and 0 % in the self-fixating mesh group, with no statistically significant differences. On the other hand, recurrency in the staple fixation group was 2.28 % versus 3.90 % in the self-fixating mesh group, without statistically significant differences.

**Conclusions:**

Self-fixating meshes have a trend towards smaller proportion of chronic pain and similar proportions of recurrence; therefore, they seem to be the best fixation method between the two mechanisms that are available in our field to prevent postoperative chronic pain.

## 1 Introduction

Hernia repair is one of the world's most frequently performed surgical procedures [1]. In the ’90s, minimally-invasive surgical management techniques were developed, including the laparoscopic TransAbdominal PrePeritoneal (TAPP) inguinal hernia repair [2]. The advantages of this approach in relation to open procedures are a lower risk of surgical wound complications, a reduction in acute and chronic postoperative pain, and an early return to activities [3]. The current recommendation for herniorrhaphy involves the placement of a mesh, which, in the TAPP technique, is placed in the extraperitoneal space; and, its minimal dimensions should be 10 × 15 cm in order to cover the myopectineal orifice [4,5].

When placing the mesh, fixation may be required to prevent migration and avoid recurrence; nevertheless, when considering the force and fixation method, we are confronted with tissue trauma and nerve damage, as they may cause chronic pain or other complications [3]. The prevalence of chronic pain ranges from 0.5 % to 6 %, and it is a preventable complication that alters quality of life [[6], [7], [8], [9]]. Therefore, different mesh fixation strategies have been designed, such as glue fixation, staple fixation, self-fixation meshes, or non-fixation [3].

The self-fixating mesh technique does not require any additional fixation material; it adheres to tissue through a micro-grip system that may generate less trauma to the tissue and thus cause less nerve damage. On the other hand, non-self-fixating meshes could require fixation using staples to keep them in their place, and furthermore, the need to close the peritoneum when using this technique; the advantage of this method could be a shorter surgical time, but it may imply a higher degree of tissue and nerve trauma when using this fixation method.

Considering the above, the aim of this study is to evaluate which of the two fixation strategies (staple fixation versus self-fixation mesh) used in our field is associated with the highest proportion of chronic postoperative pain in patients who underwent a TAPP inguinal herniorrhaphy and hernia recurrency.

## 2 Patients and methods

### 2.1 Study design

We designed an observational study with retrospective cohorts from patients that underwent a TAPP inguinal hernia repair between January 2014 and October 2021. This study was reviewed and approved by ethics committee (number DVO005 2084-CV1610). We followed the STROBE guidelines to report this study [10].

### 2.2 Patients

Patients with unilateral or bilateral inguinal hernia, who underwent the TAPP repair using self-fixation mesh or stapler fixation, were included. Patients who required open conversion, patients without postoperative follow-up of at least 1 year, and patients whose records did not include the variables of interest were excluded.

We analyzed the following variables: patient demographics, body mass index, presence of comorbidities, hernia repair history, smoking history in the 3 months prior to the surgical procedure, admission type (elective or urgent), hernia classification (following the European hernia society groin hernia classification: “The size of the hernia orifice is registered as 1 (≤1 finger), 2 (1–2 fingers) and 3 (≥3 fingers). This dimension is also reported to be identical to the length of the branches of a pair of most laparoscopic graspers, dissectors or scissors, enabling the surgeon to use the same classification during laparoscopic surgery” [11]), unilateral or bilateral hernia, associated procedures, operative time, hospital stay, complications, recurrence during follow-up and the presence of chronic pain (defined as pain lasting for 3 months after the injury, pain arising as a direct consequence of a nervous lesion or disease affecting the somatosensory system, in patients who did not suffer from groin pain before their original hernia operation, or, if they did, that this post-operative pain differed from the pre-operative pain previously experienced [7]).

### 2.3 Mesh description

When using the self-fixating mesh, it had the following characteristics: SAMMS self-gripping mesh semi absorbable (Progrip™ Mesh, Covidien, New Haven, CT, comprised of monofilament polyester and a resorbable polylactic acid (PLA) gripping system; size of 15 × 10 cm. Mesh weight of 73.0 g/m2 (before PLA resorption); 38.0 g/m2 (after PLA resorption), and its porosity (pore size: 1.1 × 1.7 mm) [1].

When the mesh was used with staple fixation, it had the following characteristics: Non-absorbable mesh (Parietene™ Macroporous Mesh, Covidien), comprised of monofilament polypropylene; size is 15 × 10 cm; mesh weight is 46 g/m2, and its porosity (2.0 × 2.4 mm).

The fixation device and the staples had the following characteristics: The AbsorbaTack™ 5 mm fixation device. The tack is constructed out of an absorbable synthetic polyester copolymer derived from lactic and glycolic acid.

### 2.4 Surgical procedure

TAPP inguinal hernia repair was performed using the 3-port standard technique. 60 to 30 min before the incision, 2 g of cefazolin were administered. One 12 mm trocar was inserted at umbilical level, and two 5 mm trocars laterally. A parietal peritoneum flap was performed from the anterior superior iliac spine to the medial umbilical ligament on the herniated side. The proposed steps for achieving the critical view of the myopectineal orifice were followed [5]; the 15 × 10 cm mesh, which was introduced through the 10 mm port at umbilical level, was then placed. In the case of non-self-fixating meshes, these were fixed with staples, avoiding the pain triangle, the peritoneum was then closed with staples; in the case of the self-fixating meshes, the parietal peritoneum flap was closed using a slow absorbing monofilament suture.

All patients received general anesthesia, by routine local anesthesia was used before each incision, and were given treatment for their acute postoperative pain with acetaminophen and non-selective COX inhibitors for 5 days. No patient received preoperative or intraoperative prophylactic treatment for chronic pain. Prophylactic neurectomies were not performed either.

### 2.5 Statistical analysis

The demographic, clinical, surgical variables, as well as the outcomes (primary: chronic postoperative pain; secondary: recurrence, complications, hospital stay and operative time) were described. Categorical variables were described as proportions and continuous variables as medians with their corresponding interquartile range (IQR). A bivariate analysis, using the chi-squared test for categorical variables and the Mann-Whitney test for continuous variables was performed between the patients with staple fixation of the mesh and those with self-fixating mesh.

The entire analysis was performed in SPSS®28, considering a *p* value < 0.05 as statistically significant.

## 3 Results

**Fig. 1:**
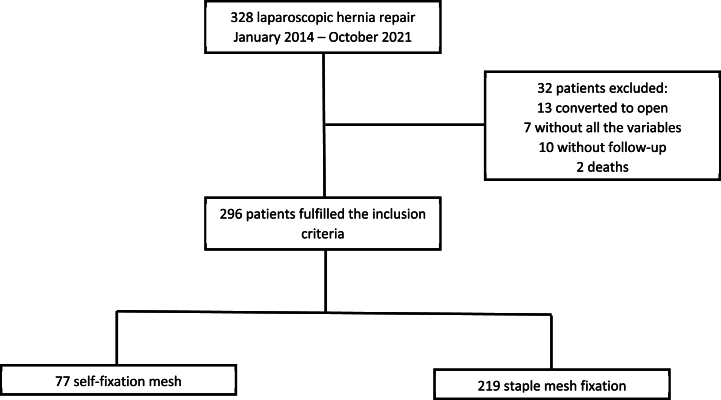
Flowchart of the study selection process.

This study included 296 patients, out of which 219 (73.48 %) had their mesh fixed with staples. The screening process is shown in the following flowchart **(**Fig. 1**)**.

Two deaths occurred: one due to abdominal sepsis caused by a strangulated hernia that required resection and anastomosis, and the other one due to healthcare-associated pneumonia; both were excluded from the analysis.

Patients’ median age was 66.0 (IQR: 20.75) years and were predominantly male (69.93 %). Table 1 shows the demographic, clinical, and surgical characteristics of patients according to mesh fixation method.

**Table 1:** Demographic, clinical and surgical characteristics according to mesh fixation method.

	N = 296 (%)	Self-fixating mesh (%) n = 77	Staple mesh fixation (%) n = 219	p value
Age (median)(IQR) years	66 (20.75)	68 (20.00)	66 (21.00)	0.373*
Sex				0.534
Female	89 (30.07)	21 (27.27)	68 (31.05)	
Male	207 (69.93)	56 (72.73)	151 (68.95)	
Body mass index (median)(IQR)(kg/m^2^)	24.9 (4.82)	24.2 (5.15)	25.2 (4.70)	0.295*
Co-morbidity				
Diabetes mellitus	24 (8.11)	8 (10.39)	16 (7.31)	0.394
Arterial hypertension	82 (27.7)	23 (29.87)	59 (26.94)	0.621
Chronic obstructive pulmonary disease	16 (5.41)	3 (3.90)	13 (5.94)	0.496
Cardiovascular disease	15 (5.07)	4 (5.19)	11 (5.02)	0.953
Immunosuppression	4 (1.35)	2 (2.60)	2 (0.91)	0.271
Chronic kidney disease	2 (0.68)	1 (1.30)	1 (0.46)	0.438
End-stage kidney disease	2 (0.68)	1 (1.30)	1 (0.46)	0.438
Corticosteroid use	8 (2.70)	0 (0.00)	8 (3.65)	0.088
Chemotherapy in the last 6 months	2 (0.68)	0 (0.00)	2 (0.91)	0.400
Groin hernia repair history	42 (14.19)	10 (12.99)	32 (14.61)	0.725
Smoking history in the 3 months prior to the surgical procedure	21 (7.09)	3 (3.90)	18 (8.22)	0.204
Admission type				0.439
Urgent	63 (21.28)	14 (18.18)	49 (22.37)	
Elective	233 (78.72)	63 (81.82)	170 (77.63)	
Size of the hernia orifice				0.170
1	70 (23.65)	16 (20.78)	54 (24.66)	
2	179 (60.47)	53 (68.83)	126 (57.53)	
3	47 (15.88)	8 (10.39)	39 (17.81)	
Laterality				**0.048**
Unilateral	144 (48.65)	30 (38.96)	114 (52.05)	
Bilateral	152 (51.35)	47 (61.04)	105 (47.95)	
Associated procedures				0.911
Umbilical hernia repair	83 (28.04)	24 (31.17)	59 (26.94)	
Ventral hernia repair	7 (2.36)	2 (2.60)	5 (2.28)	
Bowel resection	1 (0.34)	0 (0.00)	1 (0.46)	
Cholecystectomy	3 (1.01)	1 (1.30)	2 (0.91)	

*1. p* values were obtained using the chi-squared test.

2. *p values were obtained using the Mann–Whitney test.

3. Bold values indicate statistically significant *p* values (*p* < 0.05).

The proportion of participants with chronic pain was 3.20 % in the staple fixation group and 0 % in the self-fixating mesh group, without a statistically significant difference. As for recurrence, the proportion was 2.70 %, and it was higher in the self-fixating mesh group. Surgical results are shown in Table 2.

**Table 2:** Surgical results after inguinal hernia repair according to mesh fixation method.

	N = 296 (%)	Self-fixating mesh (%) n = 77	Staple mesh fixation (%) n = 219	p value
Operative time (median)(IQR) minutes	78 (44.50)	80 (52.00)	77 (45.75)	0.259*
Hospital stay (median)(IQR) days	1 (0.00)	1 (0.00)	1 (0.00)	0.130*
Complications				
Hematoma	31 (10.47)	6 (7.79)	25 (11.42)	0.372
Seroma	11 (3.72)	3 (3.90)	8 (3.65)	0.923
Surgical site infection	2 (0.68)	0 (0.00)	2 (0.91)	0.400
Recurrence				0.453
No	288 (97.30)	74 (96.10)	214 (97.72)	
Yes	8 (2.70)	3 (3.90)	5 (2.28)	
Chronic pain				0.112
No	289 (97.64)	77 (100.00)	212 (96.80)	
Yes	7 (2.36)	0 (0.00)	7 (3.20)	

*4. p* values were obtained using the chi-squared test.

5. *p values were obtained using the Mann–Whitney test.

6. Bold values indicate statistically significant *p* values (*p* < 0.05).

## 4 Discussion

This work evaluates two different mesh fixation techniques during inguinal hernia repair because they are the only available options in our field. The results for both patient groups were compared in the postoperative period for TAPP inguinal hernia repair using a self-fixating mesh and a staple fixation mesh. A higher rate of chronic pain was evidenced in the staple fixation group without a statistically significant difference (p = 0.112). As for the proportion of participants with complications, such as seroma, hematoma, or recurrence, there were no statistically significant differences. The same happened for the hospital length and surgical time variables. The lack of statistically significant differences in this study may probably be attributed to the lack of power in the sample size because there is a clear difference in the presence of chronic pain, with rates of 0 % in the self-fixating mesh group versus 3.2 % using the stapler fixation method.

In most of the descriptive variables, including demographic and clinical variables, we did not find any statistically significant difference between proportions, making both groups adequately comparable. Both groups had similar rates on the variable “Size of the hernia orifice,” which is relevant because the larger the hernia orifice, the higher the risk for recurrence; furthermore, the mesh fixation technique may affect recurrence depending on hernia size.

Prosthetic material fixation methods in inguinal hernia repair are still controversial, particularly due to their potential association with complications, such as chronic pain and hernia recurrence. Technical efforts are aimed at finding a balance between minimizing surgery trauma (especially in critical pain areas) and reducing recurrence rates. Fixation methods involve different options, such as using staples, sutures, glue, and, most recently, self-fixating meshes.

In several published systematic reviews and metanalyses, statistically significant differences have not been found concerning hernia recurrence or postoperative chronic pain in groups of patients who underwent inguinal hernia repairs using self-fixating meshes or staple fixation meshes. However, most of these studies have included patients with laparoscopic Total Extra Peritoneal (TEP) inguinal hernia repair, and their results are not necessarily extrapolatable to the TAPP technique [14,15]. We only identified one study using the TAPP technique, which did not show a difference between either option. For that reason, current inguinal hernia guidelines recommend against mesh fixation in minor defects in the M3-EHS classification when using the TEP technique, but there is no such recommendation when using the TAPP technique [3]. Based on a registry, it was found that when the TAPP technique was used, medial or combined defects were associated with higher recurrence. To reduce such recurrence, mesh fixation was required [16]. In light of current evidence, mesh fixation seems to be necessary when using the TAPP technique.

In a recent clinical trial where different types of mesh fixation were used during TAPP inguinal hernia repair (no fixation, glue fixation, and stapler fixation), the method of fixation did not influence the onset of chronic pain; and mesh placement without fixation produced the same pain and recurrence outcomes as mesh fixation techniques; nonetheless, this study had a limited sample size [12]. In a different observational study, where the mesh was fixated using either fibrin sealant or staplers during TAPP inguinal hernia repair was compared, there were no statistically significant differences in the appearance of chronic pain [13].

The apparent need for mesh fixation when using the TAPP technique has justified the current comparison debate among different fixation alternatives. Sajid et al. did not find differences in operative time, postoperative pain, complications, hospital stay, and recurrence when comparing staple versus glue fixation, although glue fixation was associated with a lower risk for developing chronic pain [17]. Meanwhile, in another metanalysis, Wang et al. recently compared the outcomes of inguinal hernia repair using self-fixating meshes versus conventional fixation methods, such as glue and staples, and found higher rates of chronic pain in the conventional fixation group using glue or staples but no difference in recurrence or other complications [18]. However, this systematic review did not distinguish between studies where the mesh was fixed using glue or staples. Only one study using staples was included, and it described that there were no statistically significant differences in chronic pain and recurrence, but there was a higher proportion of chronic pain appearance [19].

In general, current literature agrees that meshes fixed with staples are associated with a higher proportion of acute postoperative pain [20], which has also been associated with the larger number of staples used to fix the mesh [21]. A recent study that compared acute postoperative pain between self-fixating and staple fixation did not find statistically significant differences between both methods; however, chronic pain was not evaluated [22]. The technique for closing the peritoneum flap can also vary and may constitute a factor associated with postoperative pain, although in one study that evaluated if there were differences between the closure methods (suture or stapler), no differences were found in the proportion of pain [23].

Considering the aforementioned and this study's results, we consider using self-fixating meshes while performing TAPP inguinal hernia repair as the preferable option, considering its lower rates of postoperative chronic pain while maintaining comparable recurrence rates.

We acknowledge that our study limitations are its retrospective design, the absence of intraoperative characteristics, such as the number of staplers used, and the limited patient sample in the self-fixating mesh group; nevertheless, our results show a lower postoperative pain frequency when using self-fixating meshes, which seems logical, as it reduces tissue trauma, while having similar recurrence rates. However, the evidence for comparing these two fixation methods is still limited, and it is necessary to conduct more studies that provide more robust evidence regarding chronic pain and hernia recurrence between these two fixation techniques.

## 5 Conclusions

Self-fixating meshes have a trend towards a smaller proportion of chronic pain and similar proportions of recurrence; therefore, they seem to be the best fixation method between the two mechanisms that are available in our field to prevent postoperative chronic pain.

## Funding

This research did not receive any specific grant from the public, commercial, or not-for-profit funding agencies.

## Availability of data and material

The data used in the present study are available upon request to the corresponding author.

## Ethical standards

Ethical compliance with the Helsinki Declaration, current legislation on research Res. 008430-1993 and Res. 2378-2008 (Colombia), and the International Committee of Medical Journal Editors (ICMJE) were ensured under our Ethics and Research Institutional Committee (IRB) approval. Informed consent was filled out as required for the execution of this study.

## CRediT authorship contribution statement

**Carlos Eduardo Rey Chaves:** Writing – review & editing, Writing – original draft, Methodology, Investigation, Conceptualization. **Camilo Ramírez-Giraldo:** Writing – review & editing, Writing – original draft, Supervision, Formal analysis, Conceptualization. **Andrés Isaza-Restrepo:** Writing – review & editing, Writing – original draft, Methodology, Investigation. **Danny Conde Monroy:** Writing – review & editing, Methodology, Investigation, Conceptualization. **Juliana González-Tamayo:** Writing – review & editing, Formal analysis, Data curation, Conceptualization. **Daniela Ayala:** Writing – review & editing, Formal analysis, Data curation, Conceptualization. **Maria Carolina Moreno Matson:** Writing – review & editing, Formal analysis, Data curation, Conceptualization. **Jorge Navarro-Alean:** Writing – review & editing, Writing – original draft, Conceptualization.

## Declaration of competing interest

The authors declare that they have no known competing financial interests or personal relationships that could have appeared to influence the work reported in this paper.
